# Discrepancies between leg-to-leg bioelectrical Impedance analysis and computerized tomography in abdominal visceral fat measurement

**DOI:** 10.1038/s41598-017-08991-y

**Published:** 2017-08-22

**Authors:** Hsueh-Kuan Lu, Yu-Yawn Chen, Chinagwen Yeh, Chih-Lin Chuang, Li-Ming Chiang, Chung-Liang Lai, Kevin M. Casebolt, Ai-Chun Huang, Wen-Long Lin, Kuen-Chang Hsieh

**Affiliations:** 1 Sport Science Research Center, National Taiwan University of Sport, Taichung, 40404 Taiwan; 2 Department of Physical Education, National Taiwan University of Sport, Taichung, 40404 Taiwan; 3 Department of Nursing, St. Mary’s Junior College of Medicine, Nursing and Management, Yilan County, 26644 Taiwan; 4 Department of Dance, National Taiwan University of Sport, Taichung, 40404 Taiwan; 5 Department of Radiology, Jen-Ai Hospital, Taichung, 41265 Taiwan; 6 Hospitality, Recreation, and Tourism Management Department, East Stroudsburg University ofPennsylvania, East Stroudsburg, PA 18301 USA; 7 Department of Physical Medicine and Rehabilitation, Taichung Hospital, Ministry of Health and Welfare, Taichung, 40343 Taiwan; 8 Physical Education Teacher Certification Department, East Stroudsburg University of Pennsylvania, East Stroudsburg, PA 18301 USA; 9 Department of Leisure, Recreation, and Tourism Management, Tzu-Hui Institute of Technology, Pingtung, 92601 Taiwan; 10 Department of Sport Management, National Taiwan University of Sport, Taichung, 40404 Taiwan; 11 Office of Physical Education and Sport, National Chung Hsing University, Taichung, 40227 Taiwan; 12 Research Center, Charder Electronic Co., Ltd, Taichung, 41262 Taiwan

## Abstract

The aim of this study was to evaluate leg-to-leg bioelectrical impedance analysis (LBIA) using a four-contact electrode system for measuring abdominal visceral fat area (VFA). The present study recruited 381 (240 male and 141 female) Chinese participants to compare VFA measurements estimated by a standing LBIA system (VFALBIA) with computerized tomography (CT) scanned at the L4-L5 vertebrae (VFA_CT_). The total mean body mass index (BMI) was 24.7 ± 4.2 kg/m^2^. Correlation analysis, regression analysis, Bland-Altman plot, and paired sample *t*-tests were used to analyze the accuracy of the VFA_LBIA_. For the total subjects, the regression line was VFA_LBIA_ = 0.698 VFA_CT_ + 29.521, (correlation coefficient (r) = 0.789, standard estimate of error (SEE) = 24.470 cm^2^, *p* < 0.001), Lin’s correlation coefficient (CCC) was 0.785; and the limit of agreement (LOA; mean difference ±2 standard deviation) ranged from −43.950 to 67.951 cm^2^, LOA% (given as a percentage of mean value measured by the CT) was 48.2%. VFA_LBIA_ and VFA_CT_ showed significant difference (*p* < 0.001). Collectively, the current study indicates that LBIA has limited potential to accurately estimate visceral fat in a clinical setting.

## Introduction

Abdominal subcutaneous fat is located beneath the skin while visceral fat is located around internal organs and dispersed within the abdominal cavity. It is well understood that metabolic and cardiovascular diseases are closely related to visceral fat development in humans^1–3^. Individuals with the potential for developing metabolic disease would greatly benefit from accurate estimations of abdominal visceral fat or visceral fat area by providing valuable reference points, the prevention and treatment of visceral fat-related obesity more effectively^4^.

There are several simple indirect methods for estimating the amount of abdominal, or visceral, adipose tissue, such as body mass index (BMI), waist circumference (WC) waist-to-hip ratio (WHR)^5^, and dual-energy X-ray absorptiometry (DXA)^6^. Magnetic resonance imaging (MRI)^7^ and computerized tomography (CT)^8, 9^ have been shown to provide the most accurate assessments of abdominal adipose tissue content of the aforementioned methods. Although accurate in measurement, the application of MRI and CT are limited due to their high cost and complexity. On the contrary, bioelectrical impedance analysis (BIA) has been widely used in clinical settings and epidemiological studies due to its relatively convenient, safe, quick, and non-invasive technique for measuring body composition^10^. BIA evolved from conventional body fat mass to specifically estimating visceral fat area (VFA)^11, 12^. One of the current applications is placing the electrical plate directly on the abdomen or applying hand-to-foot or leg-to-leg mode BIA. When compared to the direct abdominal impedance BIA measuring device, the hand-to-foot mode or leg-to-leg mode BIA devices were widely used for estimating individual VFA due to their low cost, convenient, and prolonged developmental history^13^. The model for estimating body fat and body composition has been widely discussed, but very limited VFA verification studies have been reported.

However, to date, limited studies have been found comparing the differences of visceral adipose area between the leg-to-leg mode BIA versus established instruments such as MRI and CT scans^14, 15^. Specifically, using the leg-to-leg BIA system estimating BMI levels in a Chinese population has yet to be explored. However, the application of this convenient method is limited due to the lack of data supporting the use of BIA for estimating abdominal visceral adipose tissue^11^. The leg-to-leg BIA system for assessing different obesity levels needs to undergo a rigorous validation process for estimating VFA. Therefore, the direct measurement of visceral adipose tissue can be used as a reference point to cross-validate the results of the BIA measurements and evaluate its accuracy with abdominal visceral adipose estimation.

The present study recruited participants and measured their abdominal visceral fat area using a leg-to-leg BIA system and a CT scan at the L4-L5 vertebral region in order to investigate the accuracy and differences in BMI.

## Results

**Figure 1:**
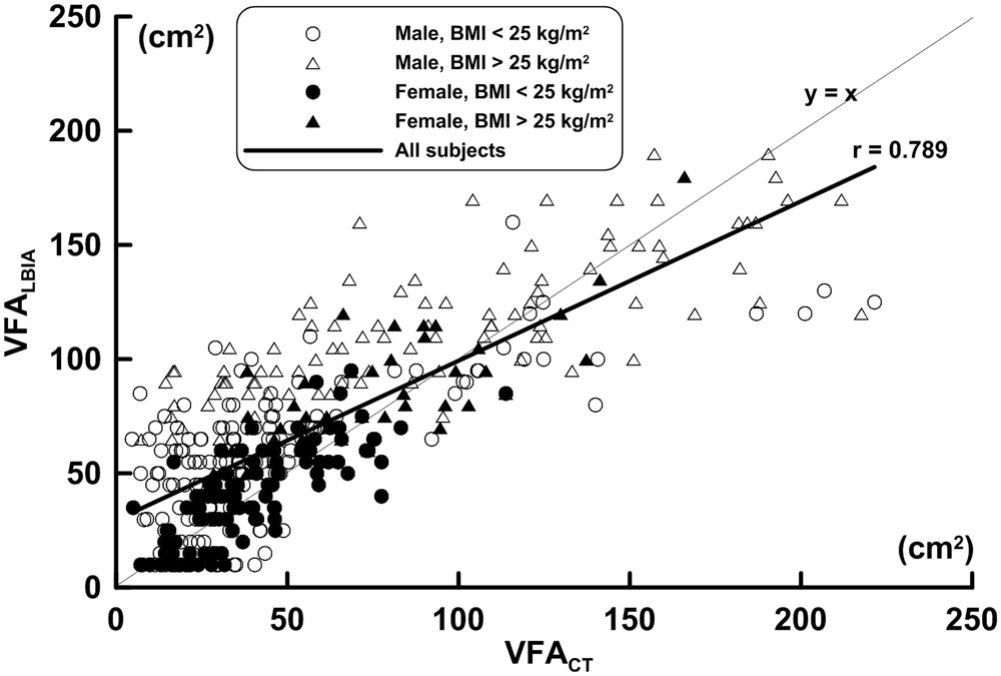
Regression analysis and scatterplot between VFA_CT_ and VFA_LBIA_ for males and females. VFA_LBIA_ = 0.698 VFA_CT_ + 29.521, *n* = 381, *p* < 0.001, *r* = 0.789, Standard estimate of error (SEE) = 24.470 cm^2^. The lines of identity and regression are shown in the figure.

Relevant anthropometric characteristics of the subjects were summarized in Table 1. These characteristics included age, body weight, height, waist circumference, hip circumference and waist-hip ratio. The VFA at L4-L5 of the vertebrae was determined by CT in female and male subjects. The correlations between VFA_CT_ and VFA_LBIA_ for the total subjects are shown in Fig. 1. The regression equation was VFA_LBIA_ = 0.698 VFA_CT_ + 29.521. The 95% confidence interval (CI) of the slope and intercept coefficient were from 0.643 to 0.752 and 25.500 to 33.542, respectively. In total, male and female subjects, the correlation coefficient and standard estimate of error (SEE) between VFA_CT_ and VFA_LBIA_ were 0.688, 0.687, 0.772 and 20.077, 21.359, 13.298 cm^2^ in subjects with BMI <25 kg/m^2^. In addition, 0.770, 0.779, 0.813 and 22.078, 19.354, 15.949 cm^2^ were reported for subjects with BMI ≧ 25 kg/m^2^. Furthermore, male and female subjects’ correlation coefficient between VFA_CT_ and age were r = 0.782 and 0.852 (*p* < 0.001). In total, male and female subjects, the concordance correlation coefficient (CCC) between VFA_CT_ and VFA_LBIA_ values were 0.784, 0.715, 0.845. Subjects that had BMI < 25 kg/m^2^ had CCC values of 0.654, 0.613, 0.770 and individuals with BMI ≥ 25 kg/m^2^ had CCC values of 0.663, 0589, 0.751, respectively.

**Table 1:** Physical characteristic and measured abdominal visceral adipose tissue of the subjects.

	**Male**	**Female**
	**Physical characteristics**	
	*n* = 240	*n* = 141
Age (year)	34.9 ± 16.5 (20.3, 81.5)*	34.2 ± 16.7 (18.8, 74.8)
Height (cm)	172.9 ± 7.6 (151.5, 197.4)**	159.6 ± 6.3 (143.0, 174.0)
Weight (kg)	74.2 ± 13.1 (45.0, 131.0)**	62.6 ± 11.1 (45.0, 109.0)
Waistline (cm)	85.2 ± 11.5 (68.2, 121.6)*	77.9 ± 10.9 (66.0, 122.2)
Hip (cm)	98.3 ± 7.2 (88.3, 120.3)	95.0 ± 8.1 (84.0, 129.4)
Waist-hip ration	0.86 ± 0.06 (0.77, 1.02)*	0.82 ± 0.06 (0.73, 1.00)
BMI (kg/m^2^)	24.8 ± 3.8 (16.3, 41.7)*	24.6 ± 4.3 (17.2, 39.1)
	**Abdominal adipose tissue (total subjects)**	
VFA_CT_ (cm^2^)	63.7 ± 51.4 (4.7, 221.5)*	48.4 ± 29.3 (4.9, 184.5)
SFA_CT_ (cm^2^)	108.2 ± 89.3 (10.1, 513.2)**	173.2 ± 100.1 (8.1, 506.2)
ACSA_CT_(cm^2^)	463.1 ± 125.9 (277.8, 981.4)	474.2 ± 136.6 (284.4, 1011.4)
VFA_CT_/ACSA_CT_ (%)	12.6 ± 8.2 (1.2, 40.5)*	9.6 ± 3.8 (1.3, 20.3)
SFA_CT_/ACSA_CT_ (%)	20.8 ± 11.4 (0.4, 53.3)**	34.0 ± 11.4 (2.3, 59.7)
VFA_LBIA_ (cm^2^)	80.5 ± 40.9 (10.0, 190.0)**	44.3 ± 26.1 (8.3, 153.0)
	**BMI** < **25** **kg/m** ^**2**^	
	*n* = 148	*n* = 87
VFA_CT_ (cm^2^)	45.1 ± 39.1 (4.7, 221.5)*	43.0 ± 22.4 (5,5, 126.4)
SFA_CT_ (cm^2^)	65.2 ± 41.8 (1.1, 207.5)**	131.3 ± 60.6 (8.1, 309.5)
ACSA_CT_(cm^2^)	396.7 ± 61.3 (277.8, 564.2)*	417.2 ± 80.0 (288.4, 648.3)
VFA_CT_/ACSA_CT_ (%)	10.7 ± 7.6 (1.2, 40.5)*	9.9 ± 3.9 (1.4, 19.5)
SFA_CT_/ACSA_CT_ (%)	15.6 ± 8.2 (0.4, 40.1)**	30.1 ± 9.4 (2.3, 50.9)
VFA_LBIA_ (cm^2^)	58.5 ± 29.3 (10.0, 160.0)**	34.0 ± 17.7 (8.3, 80.4)
	**BMI** ≥ **25** **kg/m** ^**2**^	
	*n* = 92	*n* = 54
VFA_CT_ (cm^2^)	93.6 ± 54.9 (7.4, 217.6)**	68.4 ± 32.5 (26.9, 166.1)
SFA_CT_ (cm^2^)	177.4 ± 101.4 (11.8, 513.2)**	260.9 ± 98.3 (77.5, 506.8)
ACSA_CT_ (cm^2^)	570.1 ± 129.7 (355.4, 981.4)**	591.4 ± 138.7(340.7, 1011.4)
VFA_CT_/ACSA_CT_ (%)	15.8 ± 8.2 (2.1, 35.2)*	12.5 ± 4.2 (5.6, 22.5)
SFA_CT_/ACSA_CT_ (%)	29.2 ± 10.8 (3.3, 52.3)**	43.0 ± 9.0 (20.8, 59.7)
VFA_LBIA_ (cm^2^)	116.0 ± 30.7 (65.0, 119.0)**	78.9 ± 27.1 (40.0, 180.0)

All values are means ± SD, minimum and maximum in parentheses; ^*, **^Significantly different from females (one-factor ANOVA): ^*^ *p* < 0.05, ^**^ *p* < 0.00^1^ ACSA: abdominal cross-sectional area, VFA: visceral fat area, SFA: subcutaneous fat area. Lower-case CT indicates results from CT scan; LBIA is a bioelectrical impedance analysis system with four-contact electrodes.

**Figure 2:**
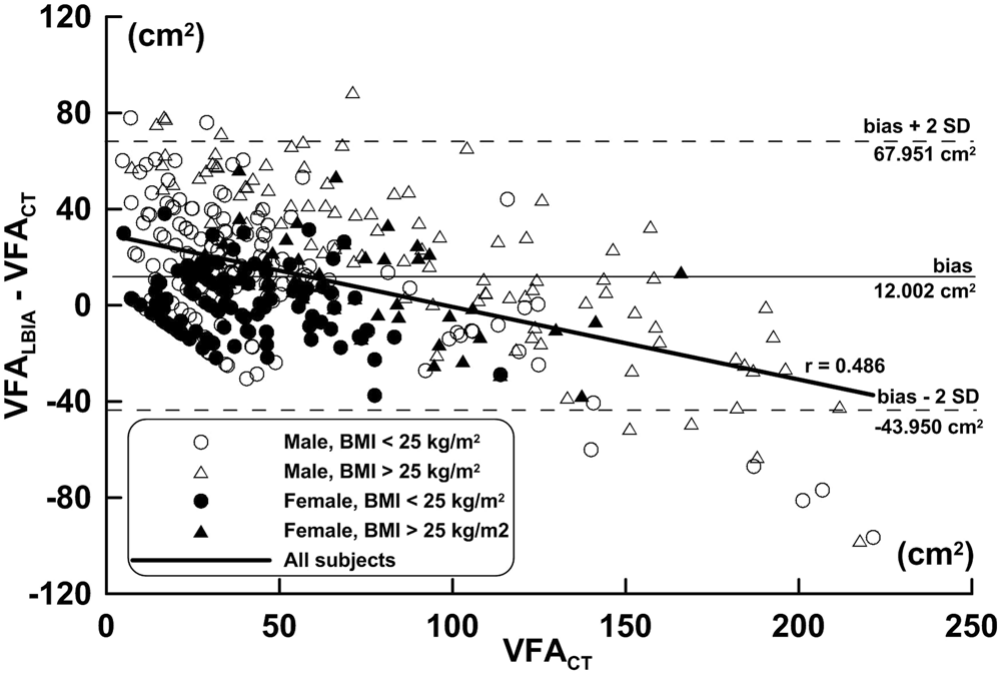
Bland-Altman plots of agreement in VFA between CT and LBIA. The horizontal solid line represents the mean difference bias between the two measuring methods. The upper and lower dotted lines represent bias ± 2 SD. The linear line is the regression line (y = −0.301 x + 29.521, *r* = 0.486, *p* < 0.001, bias = 12.002 cm^2^, bias – 2 SD = −43.950 cm^2^, bias + 2 SD = 67.951 cm^2^).

A Bland-Altman plot was used to analyze the agreement between VFA_CT_ and VFA_LBIA_ for the total participants (Fig. 2). In Fig. 2, the correlation coefficient between the x-axis and y-axis was *r* = 0.486 (*p* < 0.001). The regression line in the Bland-Altman analysis indicates that the differential value in VFA_LBIA_ and VFA_CT_ showed significant difference when VFA (X axis) was changed. In total, male and female subjects’ LOA% were 48.2%, 50.7% and 33.8%. Subjects with BMI < 25 kg/m^2^ had LOA% of 104.7%, 63.2% and 35.8% while subjects with BMI ≥ 25 kg/m^2^ were 37.6%, 38.9% and 27.7%.

**Figure 3:**
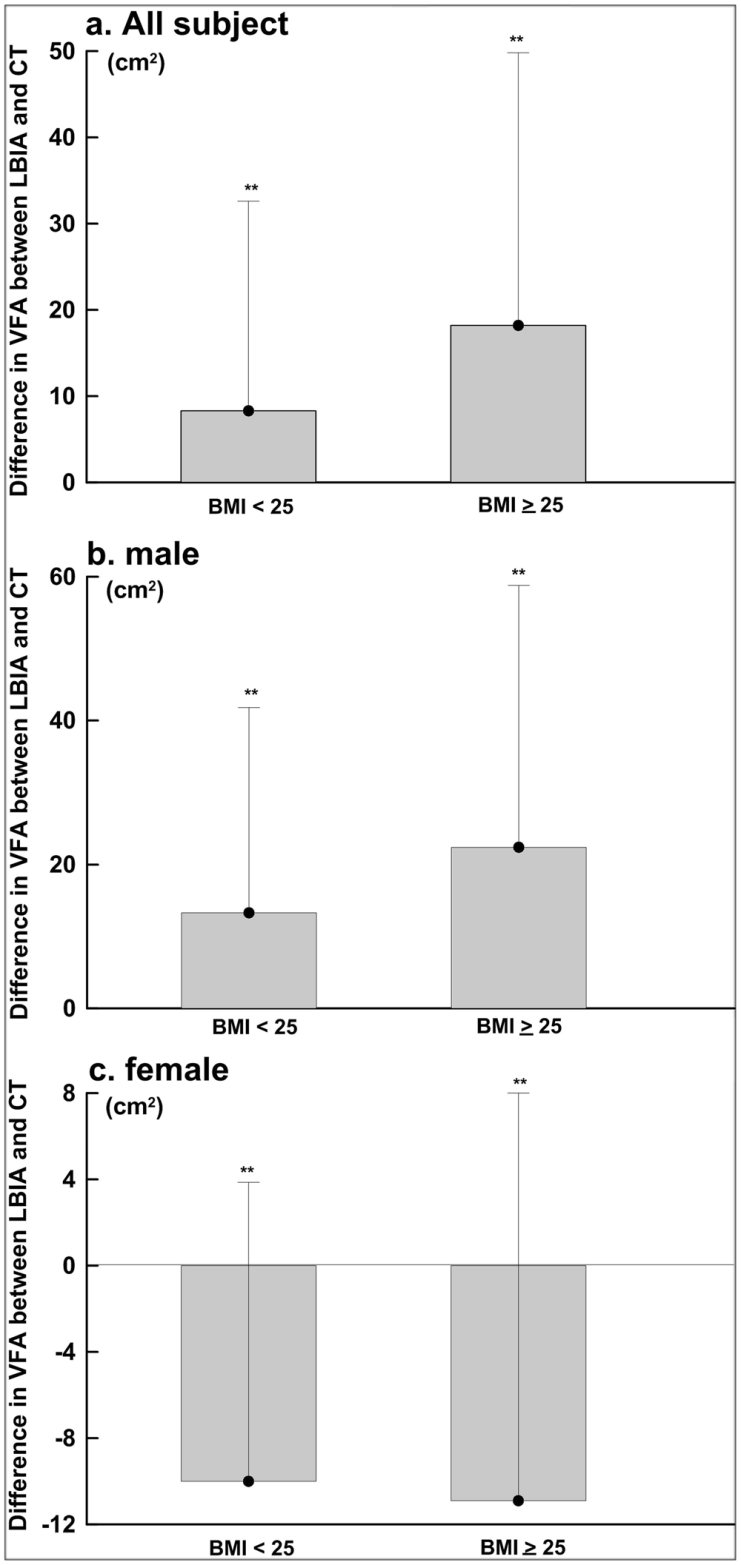
Visceral fat area (VFA) dependent bias of bioelectrical impedance analysis (VFALBIA) compared with computed tomography (VFACT) in (**a**) males and females combined (total, *n* = 381; BMI < 25 kg/m^2^, *n* = 235; BMI ≧ 25 kg/m^2^, *n* = 146), (**b**) male (total, *n* = 240; BMI < 25 kg/m^2^, *n* = 148; BMI ≧ 25 kg/m^2^, *n* = 92), and (**c**) female (total, *n* = 141; BMI < 25 kg/m^2^, *n* = 87; BMI ≧ 25 kg/m^2^, *n* = 54). Data are presented as the mean difference ±SD. Means with difference symbols are significantly different, **p < 0.001 (one-factor ANOVA).

Figures 3a–c each showed the total participants, male and female subjects’ BMI difference scores between VFA_LBIA_ and VFA_CT_. The total male and female subjects’ BMI < 25 kg/m^2^ mean values were 8.3 ± 24.3, 13.3 ± 28.5 and −10.0 ± 13.9 cm^2^ and the male and female subjects’ BMI ≧ 25 kg/m^2^ mean values were 18.2 ± 31.6, 22.4 ± 36.4 and −10.9 ± 18.9 cm^2^. In Fig. 3, a paired sample *t*-test resulted in a significant difference between VFA_LBIA_ and VFA_CT_ (*p* < 0.05). Female participants’ VFA_LBIA_ underestimated and male VFA_LBIA_ overestimated their VFA_CT_ measurements.

## Discussion

There is a pressing need for evaluating the accuracy of the leg-to-leg BIA body composition and visceral fat for individuals with respect to their home health care, but only a few studies have focused on VFA_LBIA_ accuracy assessment with a smaller sample size in the Asian population^16^. The use of regional impedance devices to assess adiposity is limited by the lack of precision and accuracy and the proprietary design^17^. However, the BIA system for body composition assessment is commercially and widely available. Therefore, the accuracy and precision of impedance devices must be assessed for their application in clinical or research settings. The present study has two distinct findings when compared to other BIA estimate studies. It used a larger sample size (381subjects) and it used Chinese participants for LBIA measurements.

To investigate measurement accuracy and precision, the impedance devices from Tanita Corp., (Tokyo, Japan) have been assessed. Bosy-Westphal *et al*.^14^ used MRI to evaluate the abdominal VFA measurement using an HBF-500 (Omron Medizintechnik, Mannheim, Germany) and a BC-532 (Tanita Europe GmbH., Sindelfingen, Germany). The correlation coefficients for the two methods in males and females were 0.82 and 0.83, respectively. Wang *et al*.^16^ used the same device to evaluate the abdominal VFA, and the correlation coefficients in males and females were 0.86 and 0.81, respectively. However, the patent for regional impedance devices used MRI to evaluate the abdominal VFA, and the correlation coefficient in males and females was stated to be 0.83 and 0.81, respectively. In the current study, different, or lower, correlation coefficients obtained from different sub-groups were caused by the participants VFACT and VFALBIA distribution. Male participants VFACT and VFALBIA were widely distributed in the BMI < 25 kg/m^2^ sub-group, hence a lower correlation coefficient. Comparatively, BMI > 25 kg/m^2^ subgroup had a higher correlation coefficient whereas the other groups showed no differences.

Using the correlation coefficient alone was insufficient to determine whether there was a relationship between the LBIA and the CT method. Thus, it was necessary to assess the magnitude of the agreement between these two methods by using a Bland- Altman Plot. Wang *et al*.^16^ studied the leg-to-leg mode of BIA-Tanita BC-532 in male and female subjects. The VFA and MRI scores were set at a 95% confidence interval with resulting scores of 20.4% (15.4% to 25.4%) and −18.0% (−24.1% to −11.9%) indicating that VFA was overestimated in males and under estimated in female subjects. This result was similar to our findings. In LBIA, the LOA for males was higher than for female individuals with underestimated VFA. Conversely, conscious measurements are considered when applying LBIA in female VFA as underestimation had occurred when transcribing the data. Scrupulous measurements are recommended when applying LBIA in female VFA as significant underestimation was found with the CT measurements. In the study conducted by Bosy-Westphal *et al*.^14^, results indicated that the LOA (LOA%) in Omron BF-500 and Tanita BC-532 was 1.9 ± 36.2 cm^2^ (45.0%) and 2.7 ± 51.8 cm^2^ (64.4%). When comparing this to LBIA 12.00 ± 55.95 cm^2^ (48.2%) of our current study, the LOA% was between that of the Omron BF-500 and Tanita BC-532 BIA devices.

In the current study, the Bland-Altman plot regression line indicated that the VFA_LBIA_ estimate would change as subject’s VFA increased or decreased. This phenomenon showed systematic bias in LBIA. In the Boy-Westphal *et al*.^14^ study, the VFA estimate using Tanita BC-532 showed a nonsignificant bias. This was a different result than the current study.

BMI is a widely used method for estimating adipose tissue in the human body and an important tool for predicting morbidity and mortality in epidemiological studies^18^. By combining the use of BMI and anthropometric parameters, we may better be able to estimate visceral adipose tissue as it becomes inadequate to measure when the level of BMI increases^19^. The most widely used WC and WHR measurements have reported in many trials that such correlation was not applicable for determining different BMI levels^20^.

VFA_LBIA_ and VFA_CT_ have positive correlations in both the different gender and BMI groups. In Fig. 1, however, the 95% CIs for the coefficients of regression slope and intercept do not cover 1.0 and 0.0, respectively, indicating that there exists a proportional bias and a fixed bias between the measurements of the two devices^21^. Also, the CCC values in VFA_LBIA_ and VFA_CT_ range from 0.614 to 0.845 for all subjects with different gender and different BMI groups. The above results indicated that the VFA estimate measuring agreement was poor in LBIA when compared with CT results. In addition, the regression line in the Bland-Altman plot showed that the VFA_LBIA_ was proportionally underestimated as subject’s VFA increased, showing that systematic bias existed in LBIA estimates. The width of the CI for the corresponding LOA is large and has a slightly larger LOA% in different gender and BMI groups. Collectively, all the above results indicated that the measuring agreement of VFA is poor in LBIA estimate in comparison with that by CT. Summary of above results and further discussion on electrophysiology, research from Danilov *et al*.^22^ and Foster *et al*.^23^ studied the sensitivity density plot and human electrical impedance distribution revealed that the leg-to-leg mode BIA would cause a short circuit from most of the trunk tissue. Due to its electrical characteristics, it cannot directly measure abdomen or visceral fat, however, the principal basis of the VFA_LBIA_ estimate is using the leg-to-leg mode BIA and anthropometry parameters to predict the weight of fat tissue. This prediction will yield a highly positive correlation between fat tissue and VFA^24^, thus validating the leg-to-leg mode and VFA correlated estimates.

When applying LBIA measuring Chinese participants’ VFA, LBIA would underestimate female and overestimate male participants VFA. As such, researchers should treat the variance of the results between males and females with caution when estimating VFA with LBIA measurements.

The current study did not measure reactance/resistance, therefore we were not able to provide further discussion on the relationship between participants’ age, gender and reactance/resistance variance. But a patent document from Shimomura *et al*.^11^ reported that leg-to-leg BIA uses height, weight, age, and fat mass to estimate VFA and the relationship for constructing the VFA estimate equation. In leg-to-leg BIA VFA measurements, if we can add in variables that have a high correlation with VFA such as age, gender or adding non-collinearity variables with reactance/resistance such as weight, sagittal diameter or subcutaneous fat thickness, after a rigorous verification process, perhaps it may increase the estimate accuracy of leg-to-leg BIA.

Chen *et al*.^25^ utilized ten subcutaneous fat thickness positions, body measurement parameters (height, weight, BMI, waist, hip, waist and hip ratio), age, and 16 other estimate variables. After applying stepwise regression analysis, waist, age, and abdominal fat area variables were used to construct the VFA estimate equation. Therefore, when estimating VFA, waist is a very important estimate variable when estimating VFA by BIA. Other than LBIA, Dual BIA is used to estimate VFA by measuring surface impedance and truncal impedance^26^. VFA values were derived from a regression formula using abdominal bioimpedance values with anthropometric parameters (abdominal transverse diameter and anteroposterior abdominal diameter) that would result in accurate VFA^27^. MRI and CT are both well-developed measuring techniques that have provided accurate reference points for VFA measurements. However, understanding how to use body composition measurements or BIA to accurately estimate VFA has become one of the main goals for researchers^28^. The authors attempted to validate the VFA measurement with CT. However, further studies are recommended to optimize different impedance measurements, such as anthropometric measures, and their relationship with VFA to obtain a better estimation of VFA^25^.

Data collected by multiple CT slices would have a better reference value than a single slice CT for calculating visceral fat tissue area. Many researchers have reported using CT or MRI measurements for a L4-L5 single slice section and reported a higher correlation with data collected by multiple CT slices^29^. Therefore, in the present study, the L4-L5 CT single slice visceral fat display area was used as a criterion reference.

The present study has several limitations. The data are limited to a Chinese population that may have different VFA characteristics than other populations. Participants’ ethnicity is mainly Asian in the current study. Therefore, the results of the current study are limited to generalizing results to American and European populations. Further, male and female participants’ lean and overweight group BMI mean were 22.49 ± 1.76, 28.33 ± 3.44 kg/m^2^ and 22.62 ± 2.18, 28.84 ± 3.85 kg/m^2^. The results of the present study are not suitable for obese group (BMI > 30 kg/m^2^) participants. Bosy-Westphal *et al*.^14^ studied a Tania BC-532 leg-to-leg BIA in a healthy German population. The correlation coefficient between VFA estimates and the reference MRI measurements showed a positive correlation but with a wide LOA. These findings were similar to the Chinese participants in the present study.

Applying leg-to-leg BIA in VFA measurements may be convenient, but in the current study, the VFA estimate results were limited in accuracy. However, but using multi-frequency or different electrode positions to measure impedance or resistance may increase the measuring accuracy for BIA in VFA^27, 30, 31^. Rigorous verification should be taken before applying it into a clinical setting environment.

However, the relatively large confidence interval between LBIA and CT raises the risk of measurement error in estimating abdominal VFA among the sample population, especially those with a BMI ≧ 25 kg/m^2^. In this study, results indicated that VFA_LBIA_ and VFA_CT_ have a positive medium correlation, large range of LOA and mean value which showed a significant difference that indicated that LBIA estimate accuracy was limited in VFA.

While BIA methods can be useful in classifying the adipose tissue distribution for the initial diagnosis of abdominal obesity for epidemiology studies^13^, the current method would have limited potential to accurately estimate visceral fat in a clinical setting.

## Methods

### Participants

381 (240 male and 141 female) Chinese subjects were recruited for this study. The participants have no major diseases, such as diabetes, cancer, kidney dysfunction, liver diseases or long-term asthma. Pregnant women, women who were likely to become pregnant, menstruating women and individuals with electronic implants (electronic devices attached to the body) were excluded from the study. Before the test, the participants had not consumed alcoholic beverages during the prior 48 hours and had not taken diuretics during the previous 7 days. Additionally, participants had not engaged in strenuous physical activity within the previous 48 hours, participants emptied their bladder 30 minutes before undergoing the LBIA measurements^32^. After the participants reported to the study site, the experimental method and procedure were explained to them by the researcher until they were fully aware of each step of the study protocol. Participants chose to participate by signing informed consent forms. The study was performed in the Radiology Department of the Dali Jen-Ai Hospital in Taichung County.

### Ethics Statement

This study was conducted according to the guidelines laid down in the Declaration of Helsinki and all procedures involving human subjects were approved by the Dali Jen-Ai Hospital ethics committee (IRB-99-02).

### Commercial leg-to-leg bioelectrical impedance analysis (LBIA) system

The abdominal visceral fat area was estimated by a single frequency (50 kHz) bioelectrical impedance analysis device (BC305, Tanita Corp., Tokyo, Japan) with tetrapolar electrodes referred to as LBIA. The height, age and gender of the participant were entered into the software system prior to the test. After the data was input into the system, the participant stood on the weight scale. The weight and electrical impedance were recorded. The VFA values were derived from a regression formula used measuring leg-to-leg impedance but the actual regression equations have not been published and a resolution of 0.5 units. The abdominal VFA measured by LBIA is referred to VFA_LBIA_ in the current study. The within-day coefficient of variation (CV%: 100 × [SD /mean]) for VFA_LBIA_ evaluated five times daily was 0.1–0.2%. The corresponding between-day CV was 0.3–0.5%.

### Computerized tomography (CT) and abdominal visceral fat area calculation

A 64-slice CT scan (Somatom Sensation 64 CT system, Siemens Corp., Germany) was performed in the abdominal region, and the data were analyzed using Software Version syngo CT2005A. Each participant lay on the center of the CT scanner while the L4-L5 vertebral region was scanned. The major CT scan parameters were the following: X-ray tube voltage 120 kV, X-ray tube electric current of 120 mA, X-ray tube collimation of 1.5 mm, rotation time of 0.5 s, slice thickness of 5 mm, slice increment of 2 mm and image reorganization smoothness index (kernel) of B20. The acquired image data were processed by the commercial software 3D-Doctor Ver. 3.5 (Able Software Corp., MA, USA). Following the protocol of a study performed by Yoshizumi^9^, the researchers scanned the umbilicus image. Additionally, the abdominal visceral adipose tissue and the abdominal subcutaneous adipose tissue were stained to calculate the fat area. Prior to calculating VFA or SFA, muscle, vascular structures, and lumbar spine area needs to be demarcated by staining the designated pixel with the same color. The CT threshold of adipose tissue range from −260 ± 3 to −10 ± 3 HU was observed from the gray scale image created from the CT scan. Measurements from the CT scan at L4-L5 of the vertebrae included the abdominal VFA, the subcutaneous fat area (SFA), and the abdominal cross-sectional area (ACSA), referred to as VFA_CT_, SFA_CT_ and ACSA_CT_, respectively.

Before the analysis, abdominal visceral fat area was measured two times with a three day interval on five participants to determine the reliability of the measurements. The correlation coefficient r and Cronbach’s alpha of the CT scans measured from the five participants’ VFA_CT_ were 0.99 and 0.99 respectively.

### Experimental procedures

The participants were asked to remove all personal metal items and to change into a lightweight cotton robe. Weight, height, waist, and hip were measured by laboratory assistant. Weight was measured to the nearest 0.1 kg (Weight-Tronix Scale, Scale Electronics Development, New York, USA). Height was measured with participants standing against a wall in their bare feet, to the nearest 0.1 cm (Stadiometer, Holtain, Crosswell, Wales, UK). Waist circumference was measured at the level of the umbilicus with both arms hanging freely while hip circumference was measured at the spina iliaca anterior superior, both were measured to the nearest centimeter. The waist circumference and the hip circumference were measured with a soft measuring tape, to the nearest 0.1 cm. The intraexaminer coefficient of variation was 3.5%.

The temperature of the test room was controlled at 25.0 ± 1.0 °C with the relative humidity (RH) set at 75%. Participants stood on four stainless steel electrodes embedded in the LBIA measurement device with their feet slightly apart to avoid inner thigh contact. Each plantar foot was in contact with a detection electrode and a current source electrode independently. The VFA estimate from LBIA was repeated three times with the mean value recorded after bioimpedance analysis. Within thirty minutes, abdominal fat was measured via CT. All of the experiments and measurements were completed from 2:00 pm to 4:00 pm.

### Statistical analysis

Data are presented as means ± standard deviations (SD). The data were analyzed using SPSS Ver. 17 (SPSS Inc., Chicago, IL, USA) and Medcal (Version 11.5; Medcalc Software, Mariakerke, Belgium). Regression analysis and paired *t*-test were performed to examine the correlation and statistical significance of mean difference observed in VFA_CT_ and VFA_LBIA_. The differences between methods (bias ± SD) and Bland -Altman analyses tested between-method agreement^33^. LOA% is given as a percentage of mean value measured by the reference method (CT). Coefficient of variation analyses determined the reliability of VFA_CT_ and VFA_LBIA_. Statistical significance was set at *p* = 0.05. The sample size of 350 for paired sample *t*-test of power analysis is determined while the effect size is 0.3, alpha value is 0.05, and power is 0.8^34^. In the study, two statistical techniques were used: the Lin’s concordance correlation (CCC) and Bland-Altman plot to assess the degree of agreement between LBIA and CT in VFA measurements^35^. The CCC was used to assess how closely the data found the line of best fit and how far the VFA measures were from the 45-degree line through the origin using LBIA and CT. The CCC and concordance scale was used with the following ratings: almost fair rating: 0.95 > CCC > 0.9; poor rating: CCC < 0.9. These ratings were used to assess the concordance of the LBIA^36^.

Furthermore, participants were regrouped according to their gender and different BMI strata for further comparison: (1) total subjects (2) male and female (3) BMI < 25 kg/m^2^ (non-overweight group) and BMI ≧ 25 kg/m^2^ (overweight and obese group).
